# From Undetectable Equals Untransmittable (U=U) to Breastfeeding: Is the Jump Short?

**DOI:** 10.3390/idr14020027

**Published:** 2022-03-25

**Authors:** Tullio Prestileo, Sanfilippo Adriana, Di Marco Lorenza, Antonina Argo

**Affiliations:** 1 Infectious Diseases Unit, ARNAS Civico Benefratelli Hospital, 90100 Palermo, Italy; prestileotullio@gmail.com (T.P.); adrianasanfilippo19@gmail.com (S.A.); 2 ANLAIDS, Sezione “Felicia Impastato”, 90100 Palermo, Italy; 3 Gastroenterology Unit, Department of Medical Specialties, University of Modena and Reggio Emilia, Azienda Ospedaliero-Universitaria di Modena, 41121 Modena, Italy; 4 Clinicaland Experimental Medicine PhD Program, University of Modena and Reggio Emilia, 41121 Modena, Italy; 5 Department of Health Promotion, Maternal and Child Care and Medical Specialties, “G. D’Alessandro”, Legal Medicine Section, University of Palermo, 90100 Palermo, Italy; antonella.argo@unipa.it

**Keywords:** HIV, pregnancy, breastfeeding, vertical transmission, freedom of choice, U=U

## Abstract

Background: Vertical transmission of HIV infection may occur during pregnancy, at childbirth or through breastfeeding. Recommendations on the safety of breastfeeding of HIV-infected women on effective antiretroviral treatment are not univocal among international guidelines (WHO 2010, EACS 2017, DHHS 2017), leaving space for variability at the patient’s level. Methods: We collected clinical, laboratory and outcome data from 13 HIV-infected pregnant women who, between March 2017 and June 2021, elected to breastfeed their children against specific medical advice. All mothers were on antiretroviral therapy with darunavir or raltegravir plus emtricitabine/tenofovir disoproxil and remained HIV-RNA undetectable and >400 cells/mmc CD4+ lymphocytes during pregnancy and breastfeeding. Prophylactic antiretroviral therapy (zidovudine for 4 weeks) was started immediately after birth in all newborns. The mean duration of breastfeeding was 5.4 months. Newborns were tested for HIV-RNA multiple times: at birth, 1, 3, and 6 months after birth, and 1, 3 and 6 months after the end of breastfeeding. Results: None of the infants were infected by HIV. Conclusions: Our experience, gathered in the setting of freedom of choice on the patient’s side, while insufficient to address the eventual safety of breastfeeding in HIV-infected mothers since the represented cohort is numerically irrelevant, supports the extension of the U=U (Undetectable Equals Untransmittable) paradigm to this setting. Since breastfeeding is often requested by women with HIV planning pregnancy, more extensive comparative studies should be performed.

## 1. Introduction

Vertical transmission of HIV infection is common in infants of HIV infected mothers, occurring during pregnancy probably through the placenta, at childbirth or through breastfeeding [1,2]. Treatment of the mother with antiretroviral therapy (ART) throughout pregnancy, delivery and possibly during breastfeeding are effective to prevent newborn infections [3,4,5,6,7]. The Promise trial [8] provided evidence of the beneficial effect on early vertical HIV transmission of maternal ART initiated during pregnancy. The trial also compared this to zidovudine (ZDV) prophylaxis in women with high CD4 counts. The French Perinatal Cohort (EPF) showed that women on ART pre-conception had the lowest risk of perinatal HIV transmission. This risk increased in the women who started ART during pregnancy [9]. Additionally, regardless of the moment of initiation of ART, the risk of vertical transmission is four times higher (95% CI 1.9–8.2) when the mother’s HIV viral load (VL) was higher than 50 copies/mL at delivery [10]. Breastfeeding, besides significant health advantages for the infant, has profound social and cultural implications, strongly felt among migrants [11]. In this vulnerable population, the problem has specific implications in the setting of HIV infection [12]. In 2010, the WHO and UNICEF issued a recommendation considering breastfeeding for women with HIV on effective ART as feasible [9]. In 2017, the European AIDS Clinical Society (EACS) reinforced this position by recommending a strict counseling plan for breastfeeding, with the aim to optimize therapeutic adherence and follow-up of mother and child [10]. However, in the US, the guidelines of the Department of Health and Human Services (DHHS) [13] have not endorsed the EACS recommendations. The DHHS clearly advised against breastfeeding, regardless of the mother’s wishes, considering a risk of vertical transmission between 0.3 and 1% [14,15]. A large body of evidence supports the efficacy of ART in preventing vertical transmission of HIV, but only 3 out of 4 of HIV positive pregnant women are on ART. Availability of effective treatment is a major challenge in underprivileged settings, with problems in access to care and in its maintenance. In fact, in specific socio-cultural contexts the risk of HIV transmission associated to breastfeeding is modest in comparison to the morbidity and mortality due to malnutrition and infections from inadequate artificial feeding, thus making breastfeeding an acceptable option [16]. Both safety issues and the right to freedom of informed choice in women on effective antiretroviral treatment who wish to breastfeed their infants remains to be verified [17]. We contribute an experience gathered dealing with HIV-infected pregnant women on effective ART who elected to breastfeed their children against specific medical advice, in order to support the possible extension of the U=U (Undetectable Equals Untransmittable) paradigm to this setting [18].

## 2. Patients and Methods

All HIV-positive pregnant women, consecutively referred between March 2017 and June 2021 to the Infectious diseases Unit, ARNAS Civico Hospital, were assessed. Clinical, laboratory and outcome data of the women and of their newborns were gathered from clinical records.

Unifying characterizing factors for enrollment of each patient in the observation cohort were:

- Being on effective antiretroviral therapy and remaining HIV-RNA undetectable and >400 cells/mmc CD4+ lymphocytes during the pregnancy and breastfeeding period
- Having been informed in multiple occasions during the pregnancy on the rules and strategies of treatment and prevention of HIV infection to the newborns, according to the WHO, EACS, and DHHS guidelines. During each visit, supported when appropriate by the presence of a cultural mediator, each patient had the opportunity to interact with the team, be able to freely discuss all the positive aspects, the risks of contagion and the importance of a precise counseling plan. The process was aimed at optimizing therapeutic adherence and close follow-up for clinical and virologic monitoring of mother and newborn
  - Having elected to breastfeed;
  - Having had a successful childbirth;
  - Having available adequate, >3 months follow up data on the newborn;
  - Giving explicit written consent to anonymous data collection.

Thirteen women and their thirteen newborns entered the observational study cohort. HIV-RNA was assessed in serum by PCR (Abbott RealTime HIV-1). Women were tested during pregnancy at week 4, 8, 12, 20, 30, at the time of delivery and every 8 weeks thereafter until the end of breastfeeding. Children were tested at birth, every 8 weeks during breastfeeding and 3 and 6 months after the conclusion of breastfeeding. Absolute and percentage values of CD3+/CD4+ and CD3+/CD8+ lymphocyte subsets and CD4+/CD8+ ratio we have used the Flow cytometry analysis (Beckman Coulter (Miami, FL, USA), Cyto-Stat^®^ Trichrome™). Immunophenotyping of specimens was performed in flow cytometry unit of Specialistic Laboratory of ARNAS Civico, G. Di Cristina and Benfratelli. Multi-Color Monoclonal Antibody (MoAb) cocktails CD45-FITC (B3821F4A clone, Ig2b mouse), CD4-PE (SFCI12T4D11 clone, IgG1 mouse), CD8-ECD (SFCI12Thy2D3 IgG1 mouse) and CD3-PC5 (UCHT1 clone IgG1 mouse) anti-human were purchased from Beckman Coulter (Miami, FL, USA). These were used according to the lyse no-wash methodology—briefly, 20 microliters MoAb were added to 100mL of peripheral blood samples incubated for 15 min at room temperature at dark and lysed for 20 min with lysing solution (Beckman Coulter, Miami, FL, USA) and, finally, acquired at NaviosTM (Beckman Coulter, Miami, FL, USA) flow cytometer. Ten thousand events for each sample, excluding debris and doublets, were acquired, and analyzed with Navios software (Beckman Coluter) CE IVD (Table 1).

## 3. Results

The mean age at delivery was 26.2 years (range 18–34). Two were native-born Italians and 11 came from African countries (four Nigeria, two Ivory Coast, two Ghana, one Mali, one Cameroon and one Senegal). African women had spent an average time of 17 months (range 6–72 months) in Italy before pregnancy. In all women, HIV infection had been acquired sexually. All women had a vaginal childbirth. Nine women, known to be infected by HIV before pregnancy, were already on ART, while four were diagnosed with HIV infection during the first 10 weeks of pregnancy and started ART immediately after diagnosis. (Table 2). Patients were treated with darunavir 8 g or raltegravir 5 g plus emtricitabine/tenofovir disoproxil 13 g daily. After the induction period of ART for the four newly diagnosed cases, all patients had undetectable HIV-RNA and a CD4 + T-Helper lymphocytes >400 cells/mmc (range 402–1560/mmc) during the entire period of pregnancy and breastfeeding (Table 3). The adherence to treatment was >95% in all patients. The mean duration of breastfeeding was 5.4 months. One woman discontinued breastfeeding permanently after 6 weeks due to SARS-CoV-2 infection. All newborns tested negative for HIV RNA at birth. Prophylactic antiretroviral therapy (zidovudine 2 mg/kg body weight orally every 6 h for 4 weeks) was started immediately after birth in all newborns. Upon retesting for HIV-RNA at 1, 3, and 6 months after birth, and 1, 3 and 6 months after the end of breastfeeding, all infants consistently tested negative for HIV infection.

## 4. Discussion

Our small cohort of carefully informed and followed pairs of mothers, mostly migrants from sub-Saharan Africa, and their offspring, is reported to focus the attention on the issue of safety of breastfeeding in HIV-infected mothers [19], especially when dealing with underprivileged and vulnerable populations. We believe that, if the “Undetectable Equals Untransmittable” (U=U) paradigm applies to sexual transmission, it should be also evaluated for other instances such as childbirth and breastfeeding where the implications of HIV transmission are intermingled with major social, cultural, ethnic, religious, and financial issues [19,20,21]. When balancing between risks and benefits in developing countries, replacement nutrition is associated with significant risks, such as an increased morbidity and infant mortality cause by diarrhea, pneumonia, and other infectious diseases [22,23,24,25,26,27]. The overall decrease of morbidity and mortality rates in breastfed infants has been attributed to protective antibodies transmitted from mother to infant through breastfeeding and decreased exposure to infectious pathogens via unsafe water [28]. We followed a group of underprivileged, mostly non-Italian mothers who, after being appropriately informed about the potential risk of HIV transmission to their offspring, accepted to start or continue their ART regimen during pregnancy, with almost absolute compliance, but elected to breastfeed their child. Eleven of the thirteen women were from Africa and had been living in Italy for a few months. All consistently stated that they wanted to breastfeed to follow the familial tradition and social education received in their country of origin. The social and cultural aspects of breastfeeding in this group of women were perceived as of major relevance. Reducing stigma related to the communication of an HIV infection of the mother, which could be necessary to motivate artificial nutrition in the patient’s microenvironment, is also a possible factor favoring breastfeeding. An important decision point were the costs, since breastfeeding, at variance with artificial nutrition, has no impact on the family’s budget. These social, cultural, and economic motivations were possibly motivating the high adherence to therapy during pregnancy and breastfeeding. Clinical and psychological support, with use of mediators to overcame language and environmental barriers, was of paramount importance to communicate appropriately the need for ART and controls and the potential risks to the offspring. Frequent checking of HIV RNA gave a feeling of empowerment to the mothers, confirming the “Undetectable” side of the U=U paradigm.

HIV infection was never detected in the offspring. Although not new, and definitely in a small-sized cohort, this fact once more confirms that an effective ART minimizes the risk of vertical transmission of HIV. As the risk of HIV transmission rises if ART is stopped during the breastfeeding period [29], the excellent compliance of mothers to their regimens throughout the observation period, together with neonatal antiretroviral prophylaxis which is effective in reducing HIV transmission in both substitute feeding and breastfeeding women [30,31,32], has probably been crucial in avoiding HIV infection in all newborns of our cohort. We feel that the most-relevant finding from our cohort is that the information available from larger studies in general populations [10,33,34,35] is also applicable, if adequately put into practice, in the setting of a vulnerable and underprivileged population in which HIV-positive mothers may live in social contexts where the pressure to breastfeed is strong. These mothers, informed in an appropriate and detailed way on the risk of contagion, should receive support that can respond to their needs through specific socio-health and psychological assistance paths [36,37]. From an ethical point of view, the autonomy of the person, here of the woman, is a fundamental and undisputed value. This concept must be compared with the principle of precaution and prudence towards the health of the child, balancing all the possible positive effects (in terms of health and well-being) in the choice to breastfeed [38]. The woman must be informed of the “predictability” of the negative effects of viral transmission.

## 5. Conclusions

In conclusion, while acknowledging the limits of a numerically limited experience, we believe that the U=U paradigm can safely be extended to the setting of breastfeeding even when the socioeconomic and cultural barriers might seem unsurmountable. Since breastfeeding is often requested by women with HIV planning pregnancy, more extensive comparative studies should be performed.

## Figures and Tables

**Table 1:** CD4 cells counts and CD4/CD8+ ratio follow-up.

CD4	CD4	CD4	CD4	CD4	CD4	CD4/CD8	CD4/CD8	CD4/CD8	CD4/CD8	CD4/CD8	CD4/CD8
Pre-Pregnancy	Week 4	Week 12	Week 20	Week 30	At the Time of Delivery	Pre-Pregnancy	Week 4	Week 12	Week 20	Week 30	At the Time of Delivery
800	756	816	906	780	1003	>1	>1	>1	0.9	1	>1
NA		402	439	509	490			0.6	0.5	0.7	0.7
535	472	518	534	516	566	0.8	0.8	0.9	1	1	0.9
671	657	599		704	680	0.7	0.7	0.7		0.6	0.8
617	599	599	680		700	0.9	>1	>1	0.9		>1
NA		500	460	523	480		0.7		0.8	0.8	0.8
402	456	416	606	480	603	0.8	1	>1	0.9	1	0.8
798	800	904	869	796	902	>1	>1	0.9	0.9	1	>1
565	572	518	594	626	666	1	0.8	>1	1	1	>1
NA	603	609	589	566	601	0.7	0.7	0.7	0.5	0.7	0.7
1006	1000	895	1021	994	1004	>1	>1	>1	1	1	>1
1560	1221	1230		1089	1218	>1	>1	>1		1	>1
NA	377	405	416	531	502		0.7	0.7	0.5	0.8	0.7

**Table 2:** Clinical, social, and demographic findings of 13 cohort female patients.

CDC	Years of Birth	Age of Delivery	Stay in Italy (Months)	Country of Origin	HIV+Diagnosis 1 (before Pregnancy) 2 (during Pregnancy)	HIV Viral Load to Delivery and during Breastfeeding	Breastfeeding Time (Months)
A-1	1999	18	6	Nigeria	1	not detected	6
A-2	1997	20	6	Mali	2	not detected	6
A-1	1987	30	48	Ivory Coast	1	not detected	11
A-1	1991	26	11	Nigeria	1	not detected	4
A-1	1993	24	6	Cameroon	1	not detected	4
A-2	1989	28	24	Ghana	2	not detected	6
A-2	1996	22	6	Ivory Coast	1	not detected	6
A-1	1985	33		Italy	1	not detected	10
A-1	1998	21	6	Nigeria	1	not detected	6
A-1	1994	34	36	Senegal	2	not detected	4
A-1	1985	33	24	Ghana	1	not detected	6
A-1	1994	28		Italy	1	not detected	6 weeks (definitive interruption for COVID-19)
A-2	1994	24	72	Nigeria	2	not detected	6

Note 1: CDC classification: Stage A-1: asymptomatic with CD4+ cell > 500/mmc, Stage A-2: asymptomatic with CD4+ cell 200–499/mm.

**Table 3:** Pre-pregnancy viral load and therapeutic scheme of 13 cohort female patients.

CDC	HIV Viral Load	Therapy
	**Pre-Pregnancy**	
A-1	not detected	lopinavir/ritonavir + emtricitabine/tenofovir
A-2	**not evaluable**	raltegravir + emtricitabine/tenofovir
A-1	not detected	lopinavir/ritonavir + emtricitabine/tenofovir
A-1	not detected	lopinavir/ritonavir + emtricitabine/tenofovir
A-1	not detected	lopinavir/ritonavir + emtricitabine/tenofovir
A-2	**not evaluable**	raltegravir + emtricitabine/tenofovir
A-2	not detected	lopinavir/ritonavir + emtricitabine/tenofovir
A-1	not detected	lopinavir/ritonavir + emtricitabine/tenofovir
A-1	not detected	lopinavir/ritonavir + emtricitabine/tenofovir
A-1	**not evaluable**	raltegravir + emtricitabine/tenofovir
A-1	not detected	lopinavir/ritonavir + emtricitabine/tenofovir
A-1	not detected	lopinavir/ritonavir + emtricitabine/tenofovir
A-2	**not evaluable**	raltegravir + emtricitabine/tenofovir
**not evaluable: HIV diagnosis during pregnancy**		

## Data Availability

Data supporting reported results can be found at the Infectious diseases Unit, 2 Migrants reception service, Civic Hospital, Palermo, Italy.
